# Supplementary material for: An NS-segment exonic splicing enhancer regulates influenza A virus replication in mammalian cells
Source: Nat Commun. 2017 Mar 21;8:14751. doi: 10.1038/ncomms14751 (PMC5364394; doi:10.1038/ncomms14751)
Supplement: Supplementary Information — Supplementary Figures and Supplementary Table [file ncomms14751-s1.pdf]

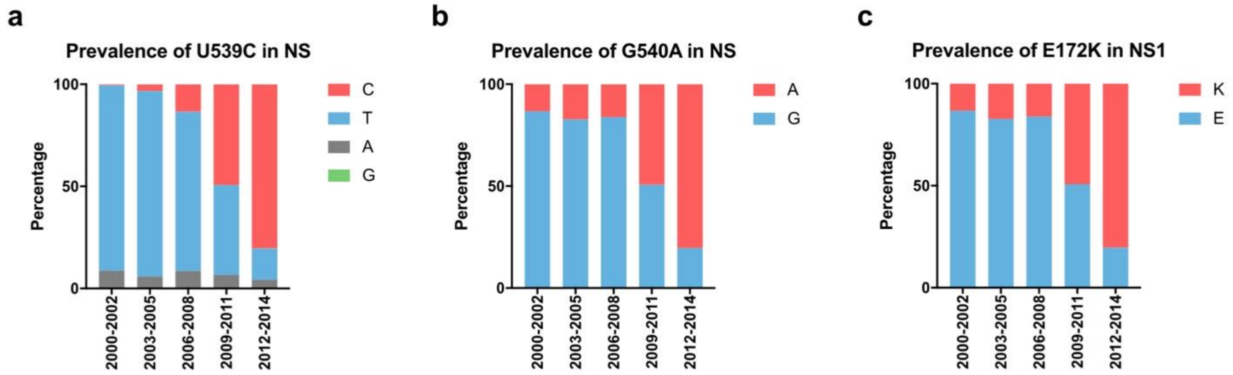

Supplementary Figure 1. Prevalence of U539C and G540A nucleotide and E172K amino acid substitutions among H9N2 viruses. Full-length H9N2 NS nucleotide sequences **(a, b)** or amino acid sequences **(c)** from the NCBI Influenza Virus Resource Database were classified and analyzed, and are presented in order of isolation, arranged into 3-year groupings.

a

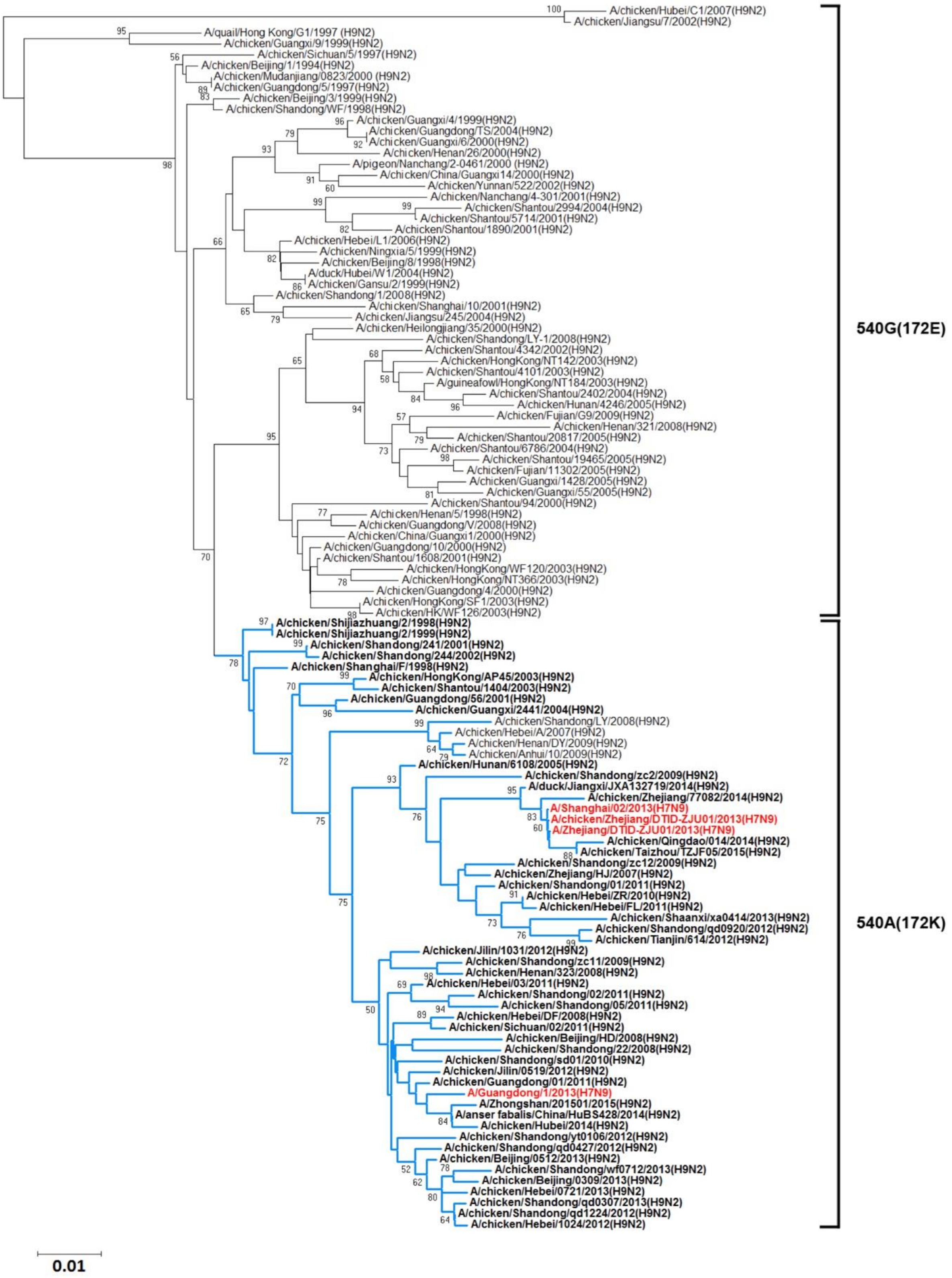

A/chicken/Hubei/C1/2007(H9N2)
A/chicken/Jiangsu/7/2002(H9N2)
A/quail/Hong Kong/G1/1997 (H9N2)
A/chicken/Guangxi/9/1999(H9N2)
A/chicken/Sichuan/5/1997(H9N2)
A/chicken/Beijing/1/1994(H9N2)
A/chicken/Mudanjiang/0823/2000 (H9N2)
A/chicken/Guangdong/5/1997(H9N2)
A/chicken/Beijing/3/1999(H9N2)
A/chicken/Shandong/WF/1998(H9N2)
A/chicken/Guangxi/4/1999(H9N2)
A/chicken/Guangdong/TS/2004(H9N2)
A/chicken/Guangxi/6/2000(H9N2)
A/chicken/Henan/26/2000(H9N2)
A/pigeon/Nanchang/2-0461/2000 (H9N2)
A/chicken/China/Guangxi14/2000(H9N2)
A/chicken/Yunnan/522/2002(H9N2)
A/chicken/Nanchang/4-301/2001(H9N2)
A/chicken/Shantou/2994/2004(H9N2)
A/chicken/Shantou/5714/2001(H9N2)
A/chicken/Shantou/1890/2001(H9N2)
A/chicken/Hebei/L1/2006(H9N2)
A/chicken/Ningxia/5/1999(H9N2)
A/chicken/Beijing/8/1998(H9N2)
A/duck/Hubei/W1/2004(H9N2)
A/chicken/Gansu/2/1999(H9N2)
A/chicken/Shandong/1/2008(H9N2)
A/chicken/Shanghai/10/2001(H9N2)
A/chicken/Jiangsu/245/2004(H9N2)
A/chicken/Heilongjiang/35/2000(H9N2)
A/chicken/Shandong/LY-1/2008(H9N2)
A/chicken/Shantou/4342/2002(H9N2)
A/chicken/HongKong/NT142/2003(H9N2)
A/chicken/Shantou/4101/2003(H9N2)
A/guineafowl/HongKong/NT184/2003(H9N2)
A/chicken/Shantou/2402/2004(H9N2)
A/chicken/Hunan/4246/2005(H9N2)
A/chicken/Fujian/G9/2009(H9N2)
A/chicken/Henan/321/2008(H9N2)
A/chicken/Shantou/20817/2005(H9N2)
A/chicken/Shantou/6786/2004(H9N2)
A/chicken/Shantou/19465/2005(H9N2)
A/chicken/Fujian/11302/2005(H9N2)
A/chicken/Guangxi/1428/2005(H9N2)
A/chicken/Guangxi/55/2005(H9N2)
A/chicken/Shantou/94/2000(H9N2)
A/chicken/Henan/5/1998(H9N2)
A/chicken/Guangdong/V/2008(H9N2)
A/chicken/China/Guangxi1/2000(H9N2)
A/chicken/Guangdong/10/2000(H9N2)
A/chicken/Shantou/1608/2001(H9N2)
A/chicken/HongKong/WF120/2003(H9N2)
A/chicken/HongKong/NT366/2003(H9N2)
A/chicken/Guangdong/4/2000(H9N2)
A/chicken/HongKong/SF1/2003(H9N2)
A/chicken/HK/WF126/2003(H9N2)
A/chicken/Shijiazhuang/2/1998(H9N2)
A/chicken/Shijiazhuang/2/1999(H9N2)
A/chicken/Shandong/241/2001(H9N2)
A/chicken/Shandong/244/2002(H9N2)
A/chicken/Shanghai/F/1998(H9N2)
A/chicken/HongKong/AP45/2003(H9N2)
A/chicken/Shantou/1404/2003(H9N2)
A/chicken/Guangdong/56/2001(H9N2)
A/chicken/Guangxi/2441/2004(H9N2)
A/chicken/Shandong/LY/2008(H9N2)
A/chicken/Hebei/A/2007(H9N2)
A/chicken/Henan/DY/2009(H9N2)
A/chicken/Anhui/10/2009(H9N2)
A/chicken/Hunan/6108/2005(H9N2)
A/chicken/Shandong/zc2/2009(H9N2)
A/duck/Jiangxi/JXA132719/2014(H9N2)
A/chicken/Zhejiang/77082/2014(H9N2)
A/Shanghai/02/2013(H7N9)
A/chicken/Zhejiang/DTID-ZJU01/2013(H7N9)
A/Zhejiang/DTID-ZJU01/2013(H7N9)
A/chicken/Qingdao/014/2014(H9N2)
A/chicken/Taizhou/TZJF05/2015(H9N2)
A/chicken/Shandong/zc12/2009(H9N2)
A/chicken/Zhejiang/HJ/2007(H9N2)
A/chicken/Shandong/01/2011(H9N2)
A/chicken/Hebei/ZR/2010(H9N2)
A/chicken/Hebei/FL/2011(H9N2)
A/chicken/Shaanxi/xa0414/2013(H9N2)
A/chicken/Shandong/qd0920/2012(H9N2)
A/chicken/Tianjin/614/2012(H9N2)
A/chicken/Jilin/1031/2012(H9N2)
A/chicken/Shandong/zc11/2009(H9N2)
A/chicken/Henan/323/2008(H9N2)
A/chicken/Hebei/03/2011(H9N2)
A/chicken/Shandong/02/2011(H9N2)
A/chicken/Shandong/05/2011(H9N2)
A/chicken/Hebei/DF/2008(H9N2)
A/chicken/Sichuan/02/2011(H9N2)
A/chicken/Beijing/HD/2008(H9N2)
A/chicken/Shandong/22/2008(H9N2)
A/chicken/Shandong/sd01/2010(H9N2)
A/chicken/Jilin/0519/2012(H9N2)
A/chicken/Guangdong/01/2011(H9N2)
A/Guangdong/1/2013(H7N9)
A/Zhongshan/201501/2015(H9N2)
A/anser fabalis/China/HuBS428/2014(H9N2)
A/chicken/Hubei/2014(H9N2)
A/chicken/Shandong/yt0106/2012(H9N2)
A/chicken/Shandong/qd0427/2012(H9N2)
A/chicken/Beijing/0512/2013(H9N2)
A/chicken/Shandong/wf0712/2013(H9N2)
A/chicken/Beijing/0309/2013(H9N2)
A/chicken/Hebei/0721/2013(H9N2)
A/chicken/Shandong/qd0307/2013(H9N2)
A/chicken/Shandong/qd1224/2012(H9N2)
A/chicken/Hebei/1024/2012(H9N2)
540G(172E)
540A(172K)
0.01

b

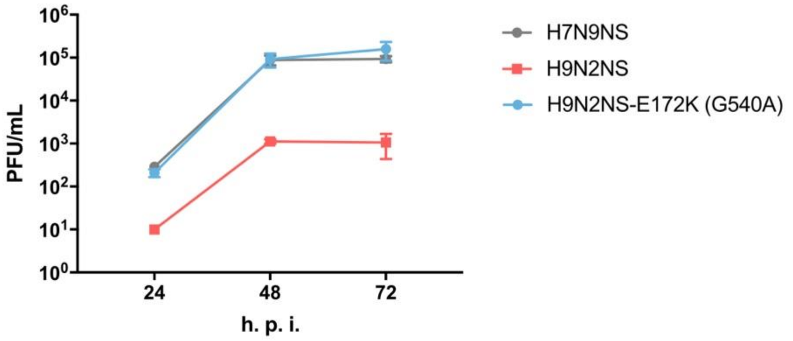

c

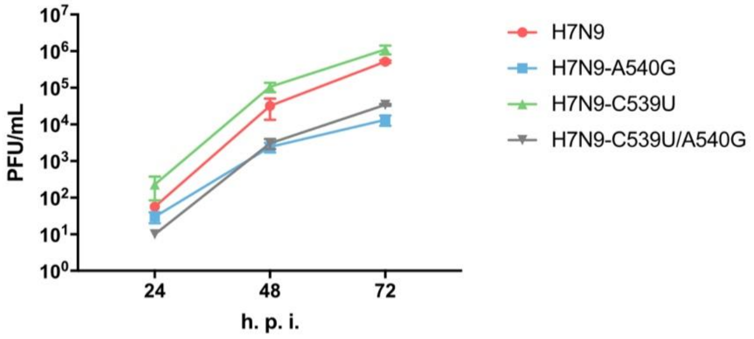

Supplementary Figure 2. (a) Phylogenetic tree of NS genes from H9N2 and H7N9. The tree includes H9N2 viruses isolated between 1994 and 2013, plus representative H7N9 viruses. Isolates carrying the 540A (172K) mutation are in boldface, and H7N9 viruses are shown in red. Node branches highlighted in blue indicate clades with the 540A (172K) mutation, except for a few that are 540G (172E). Trees were constructed using the neighbour-joining method with the Tamura-Nei model of nucleotide substitution in the MEGA program (version 5.05). Numbers below or above branches represent the bootstrap value (percentages) from 1000 replicates, to evaluate the reliability of the phylogenetic tree. **(b)** G540A nucleotide substitution in NS segment enhances replication of virus carrying H9N2-NS in human lung carcinoma cell line**.** Reassortant viruses containing WSN backbones (PB2, PB1, PA, HA, NP, NA and M) and NS from H7N9 virus or NS (WT or G540A) derived from H9N2 virus (A/Hong Kong/3239/2008, accession no: CY055160) were rescued by reverse genetics techniques. A549 cells were infected with these RG viruses at an MOI of 0.01. Supernatants were harvested at the indicated time points and the viruses present in supernatants titrated by plaque assay. The error bars represent mean ± SD (n = 3). **(c)** A540G, but not C539U, nucleotide substitutions in NS segment cause attenuated replication of virus in human lung carcinoma cell line**.** Reassortant viruses containing WSN backbones (PB2, PB1, PA, HA, NP, NA and M) and NS (WT or mutant, as indicated) derived from H7N9 virus were rescued by reverse genetics techniques. A549 cells were infected with these RG viruses at a multiplicity of infection (MOI) of 0.01. Supernatants were harvested at the indicated time points and the viruses present in supernatants titrated by plaque assay. The error bars represent mean ± SD (n = 3).

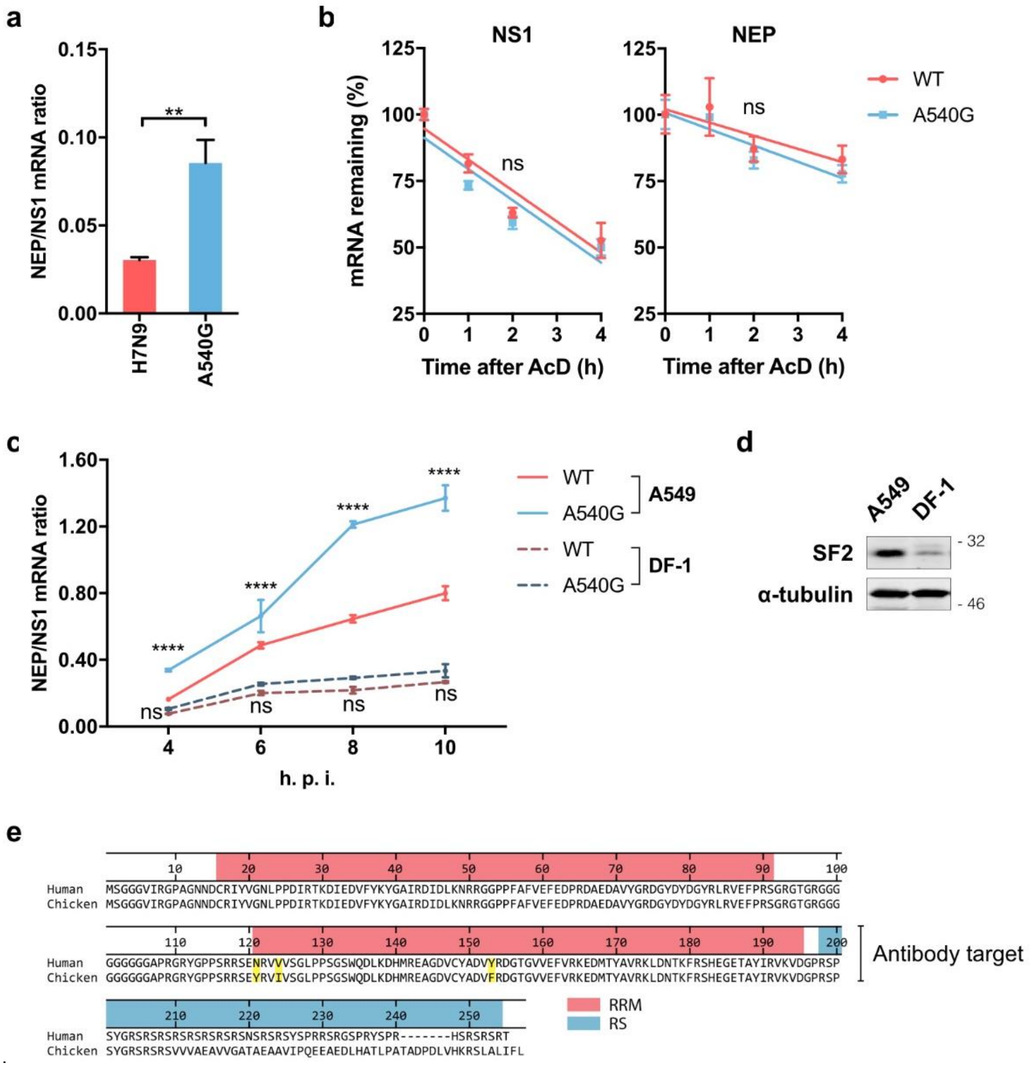

Supplementary Figure 3. G540A substitution in the NS gene ESE affects the ratio of NS1 to NEP mRNAs without changing RNA stability. (a) Total RNAs of HEK293T cells transfected with pHW2000-H7N9-NS or pHW2000-H7N9-A540G were isolated and analyzed by RT-qPCR for the ratio of NEP to NS1 mRNA. The error bars represent mean ± SD (n=3). Statistical significance was analyzed by the Student's t-test: ** $p<0.01$. **(b)** A549 cells infected with rH9N2-WT or rH9N2-NS-A540G at an MOI of 5 were treated with 10 μg/mL Actinomycin D (AcD) at 5 hr post-infection. Total RNA was extracted at indicated time points after AcD treatment. The abundance of NS1 and NEP mRNAs was measured by RT-qPCR and normalized to that of Rpl32 mRNA. Linear regression was used to compare the slopes. The error bars represent mean ± SD (n=3). ns=not significant. **(c)** Total RNA of A549 or DF-1 cells infected with rH9N2-WT or rH9N2-NS-A540G viruses at an MOI of 1 was isolated and analyzed by RT-qPCR to determine the ratio of NEP to NS1 mRNA. The error bars represent mean ± SD (n=3). Statistical significance was analyzed by two-way ANOVA with Tukey's multiple comparisons test: **** $p<0.0001$, ns=not significant. h.p.i. - hours post-infection. **(d)** Whole cell lysates of A549 and DF-1 cells were analyzed by immunoblotting with polyclonal Abs against endogenous SF2 and α-tubulin. **(e)** Illustration of the sequence alignment and immunogenic target of the polyclonal Ab against SF2 used in (d).

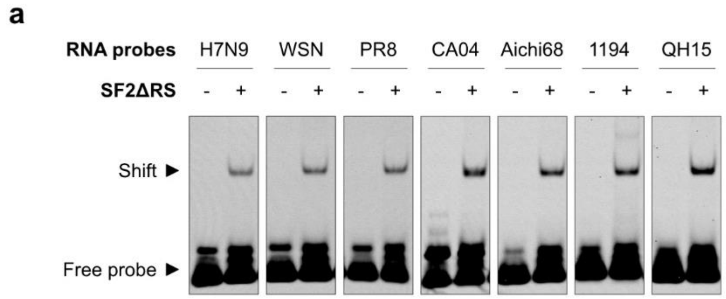

Strains	RNA probes (20 mer)	Scores
H7N9	GACAUACUGACAAGGAUGUC	2.64
H1N1 (WSN)	GACAUACUGAUGAGGAUGUC	2.70/2.11
H1N1 (PR8)	GACAUACUGCUGAGGAUGUC	4.62
H1N1 (CA04)	GACAUACUUAUGAGGAUGUC	2.11
H3N2 (Aichi68)	GACAUACUAUUGAGGAUGUC	2.16
H5N1 (1194)	GACAUACUGGUGAGGAUGUC	3.04
H5N1(QH15)	GACAUACUAAUGAGGAUGUC	2.16/2.11

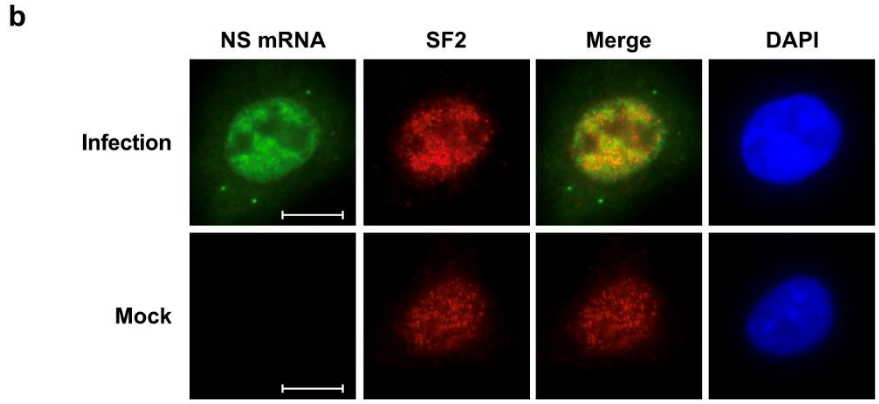

Supplementary Figure 4. (a) SF2-NEP ESE binding is a universal mechanism among influenza A viruses.

(Upper Panel) RNA probes spanning putative ESE motifs from various strains, as indicated, were shifted by SF2ΔRS in RNA EMSA. (Lower Panel) Sequences of RNA probes for different influenza A virus strains. Putative ESE motifs in the different strains were predicted using the SF2/ASF matrix of the ESEfinder program, with a threshold of 1.956, and are marked in red (highest SF2-ESE motif score) or underlined (lower SF2-ESE motif score, if applicable). The scores are listed. (b) NS mRNA and SF2/ASF co-localize in the nucleus. A549 cells infected with H9N2-WT viruses at an MOI of 5 were fixed at 8 hr post-infection. Co-localization of NS mRNA (green) and SF2/ASF (endogenous, red) was analyzed by sequential immunofluorescence (IF) with α-SF2/ASF and fluorescence in situ hybridization (FISH) with RNA probes against NS mRNA. Images were captured using a wide field microscope. Scale bar, 10 μm.

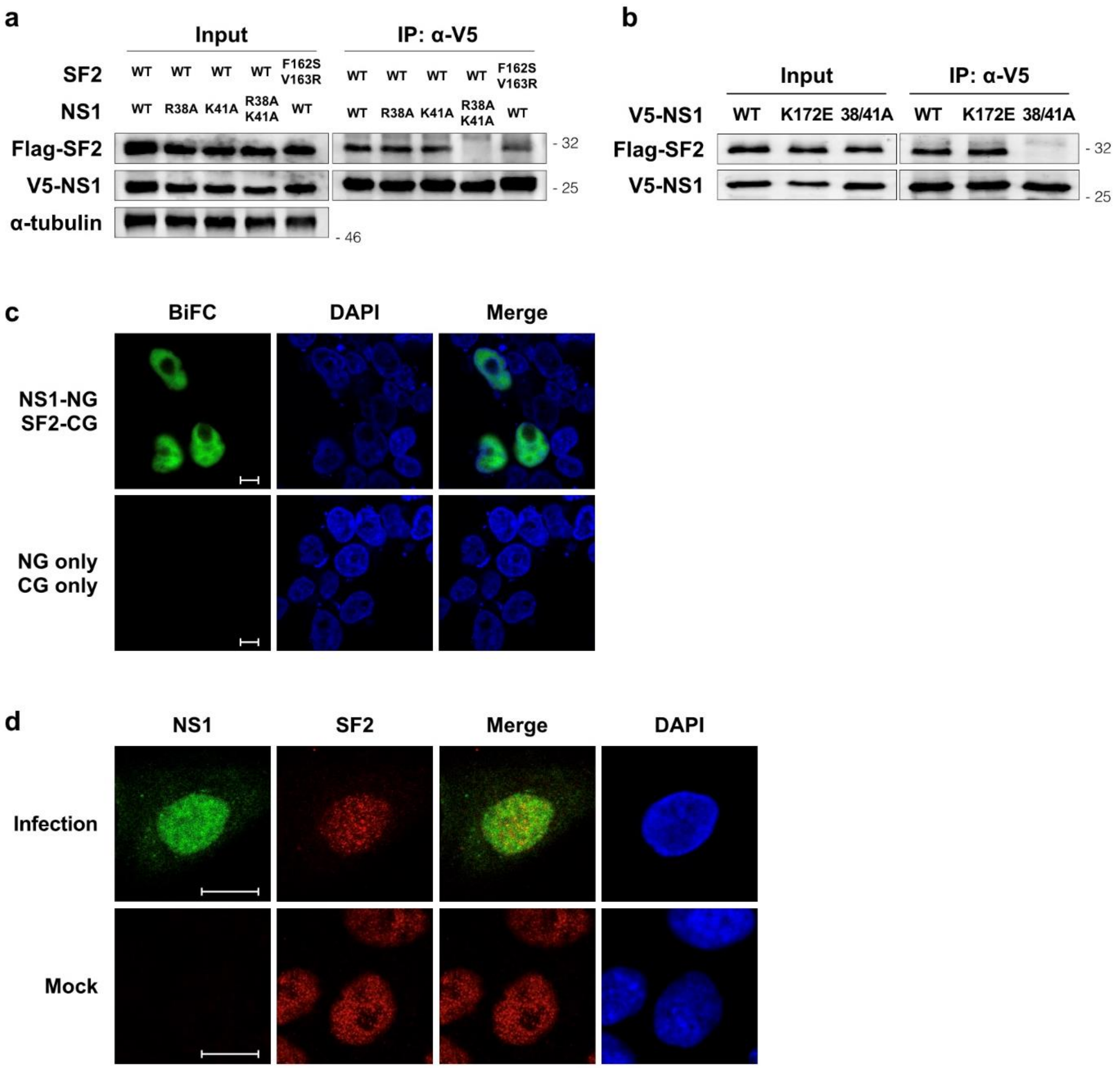

Supplementary Figure 5. (a) NS1-R38A/K41A mutant cannot interact with SF2. Whole cell lysates of HEK293T cells transfected with FLAG-SF2 or FLAG-SF2-F162S/V163R, together with V5-NS1, V5-NS1-R38A, V5-NS1-K41A or V5-NS1-R38A/K41A were subjected to IP with α-V5, followed by immunoblotting with Abs against FLAG, V5 and α-tubulin. (b) K172E does not affect the interaction between SF2 and NS1. Whole cell lysates of HEK293T cells transfected with FLAG-SF2 and V5-NS1, V5-NS1-K172E or V5-NS1-R38A/ K41A (38/41A) were subjected to IP with α-V5, followed by IB with Abs against FLAG and V5. (c) Subcellular localization of the BiFC NS1-SF2 complex. HEK293T cells were transfected with NS1-NG/SF2-CG or NG/CG combinations of plasmids for BiFC. Cells were fixed at 24 hr post-transfection and stained with DAPI. Images were acquired by confocal microscopy. Scale bar, 10 μm. (d) NS1 co-localizes with SF2/ASF in the nucleus. A549 cells infected or mock infected with H9N2-WT viruses at an MOI of 5 were fixed at 6 hr post-infection, followed by IF with α-SF2/ASF (red) and α-NS1 (green). Images were acquired by confocal microscopy. Scale bar, 10 μm.

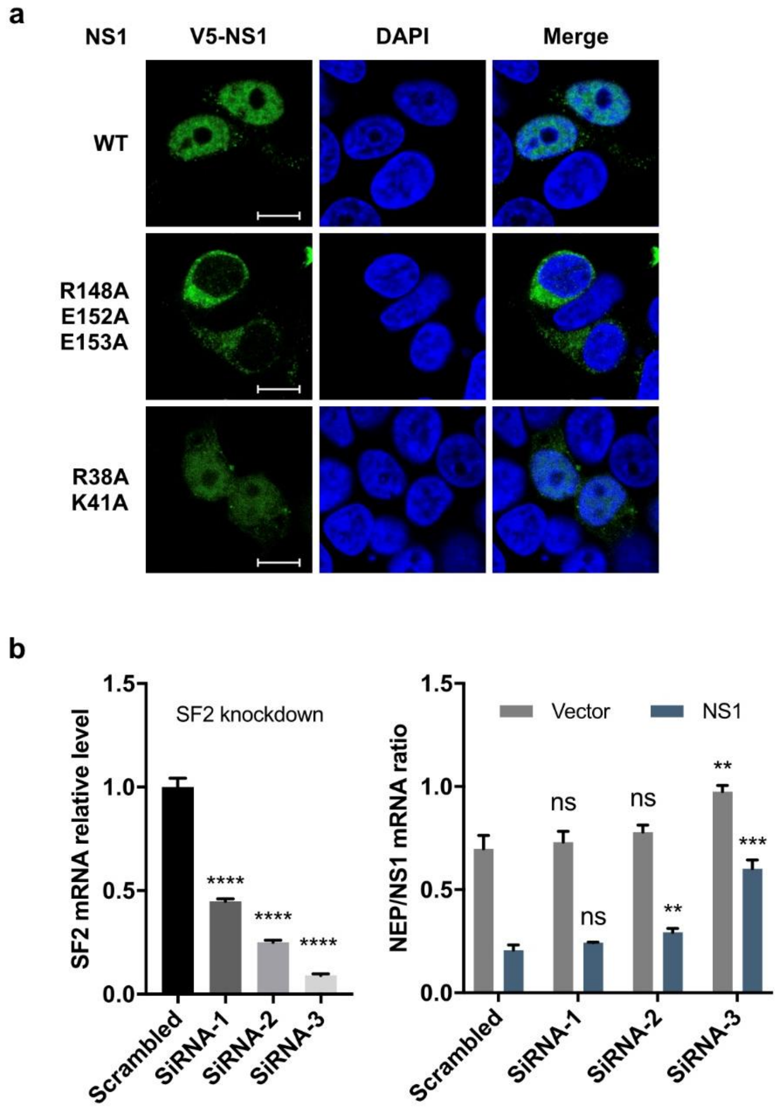

Supplementary Figure 6. Cellular localization of NS1 mutants and effects of the NS1 protein on NEP/NS1 mRNA ratio in SF2 knockdown cells. (a) HEK293T cells were transfected for 16 hr with pHI-NS-null plasmid and RNP plasmids, together with V5-NS1, V5-NS1-R38A/K41A or V5-NS1-R148A/E152A/E153A. Cells were fixed at 16 hr post-transfection. The locations of the indicated NS1 proteins were analyzed by IF with α-V5 (green). Images were acquired by confocal microscopy. Scale bar, 10 μm. (**b**) HEK293T cells were transfected with siRNAs against SF2 or scrambled siRNA as a negative control. (Left panel) SF2 mRNA abundance was analyzed by RT-qPCR. The error bars represent mean ± SD (n=6). (Right panel) SF2 knockdown HEK293T cells were transfected with pHI-NS-null and RNP, together with empty vector or NS1 expression plasmid. Total RNA was isolated and analyzed by RT-qPCR to determine the ratio of NEP to NS1 mRNA. The error bars represent mean ± SD (n=3). The statistical significance of the difference between knockdown and scrambled was analyzed by the Student's t-test: ** $p<0.01$, *** $p<0.001$, **** $p<0.0001$, ns=not significant.

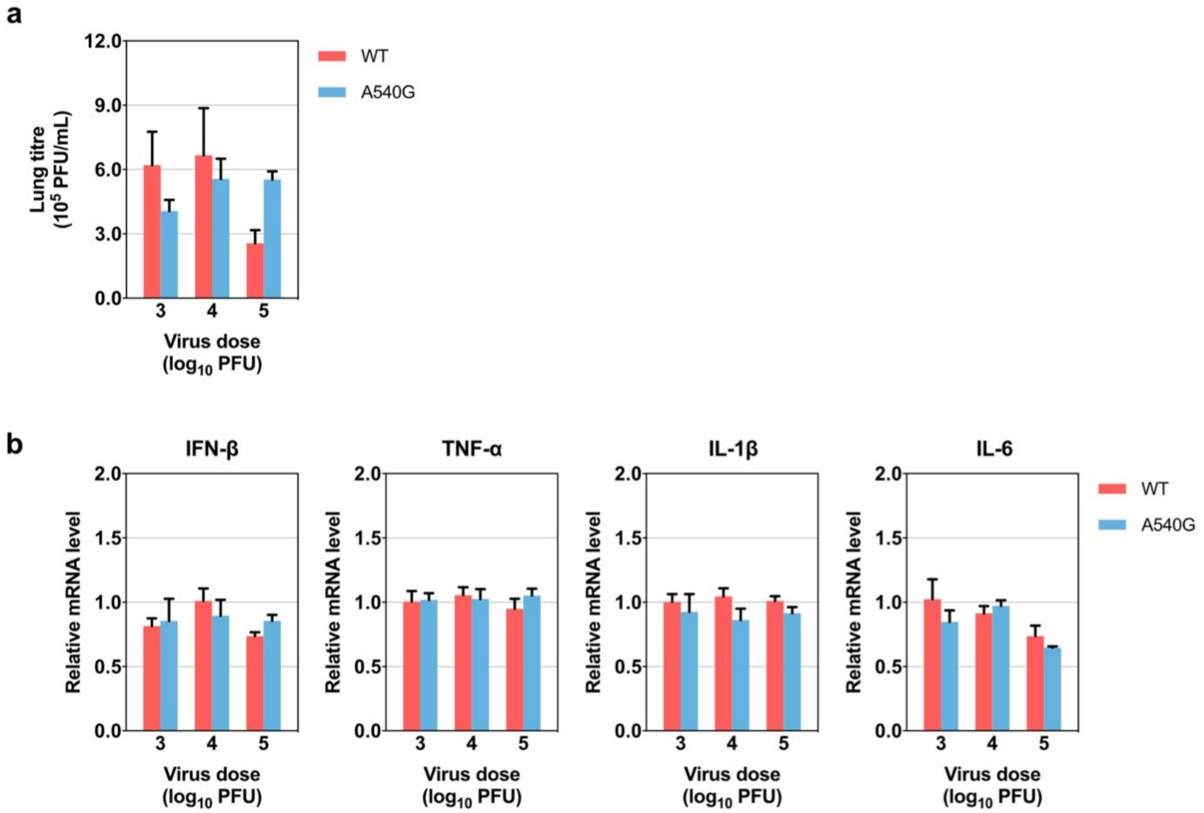

Supplementary Figure 7. (a) Titration of virus titers in lung tissues from WT or NS-A540G mutant H7N9 virus infected mice. Mice were challenged with different doses of either WT or NS-A540G mutant H7N9 virus. On day 3 post infection, 3 mice were sacrificed and lung tissues collected. Virus titers were estimated by plaque assay, as described in the materials and methods. (b) Levels of cytokine mRNA in lung tissues from infected mice. RNAs were extracted from the lung tissue homogenates collected in S7a. Levels of IFN-β, TNF-α, IL-1β and IL-6 mRNA were detected by RT-qPCR and normalized to that of mouse GAPDH mRNA. The error bars represent mean ± SD (n=3).

Fig. 2a

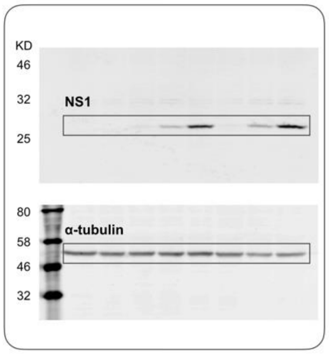

Fig. 2b

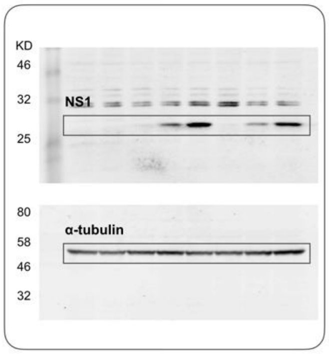

Fig. 2g

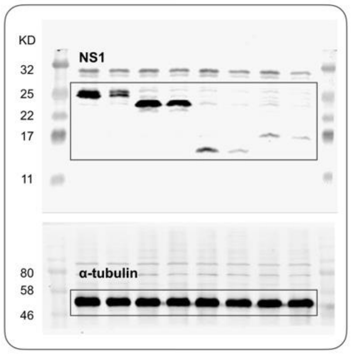

Fig. 2h

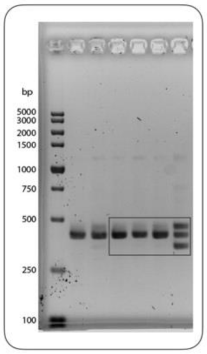

Fig. 3a

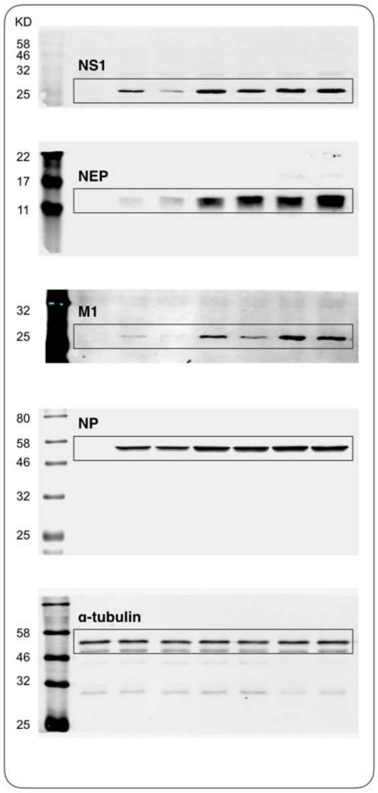

Fig. 4b

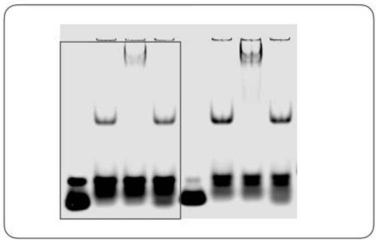

Fig. 4c

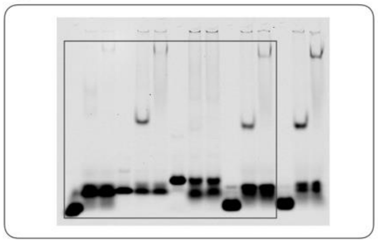

Fig. 4d

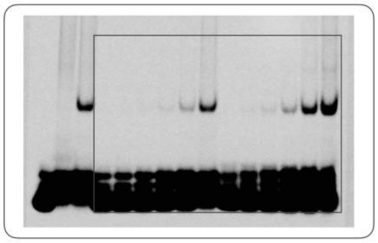

Fig. 4e

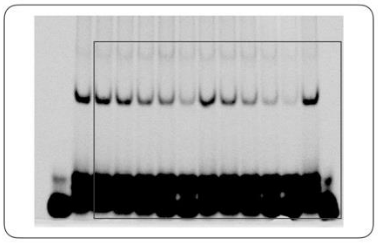

Fig. 4f

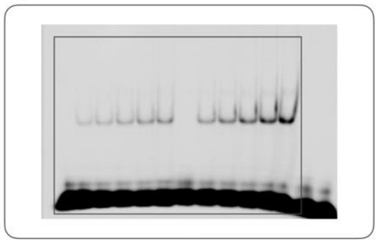

Fig. 5a

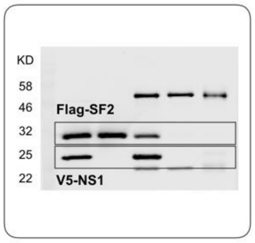

Fig. 5b

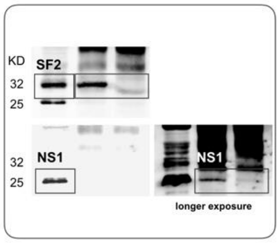

Fig. 5c

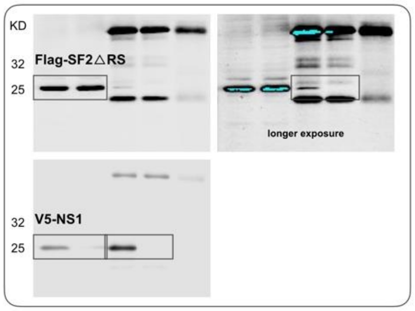

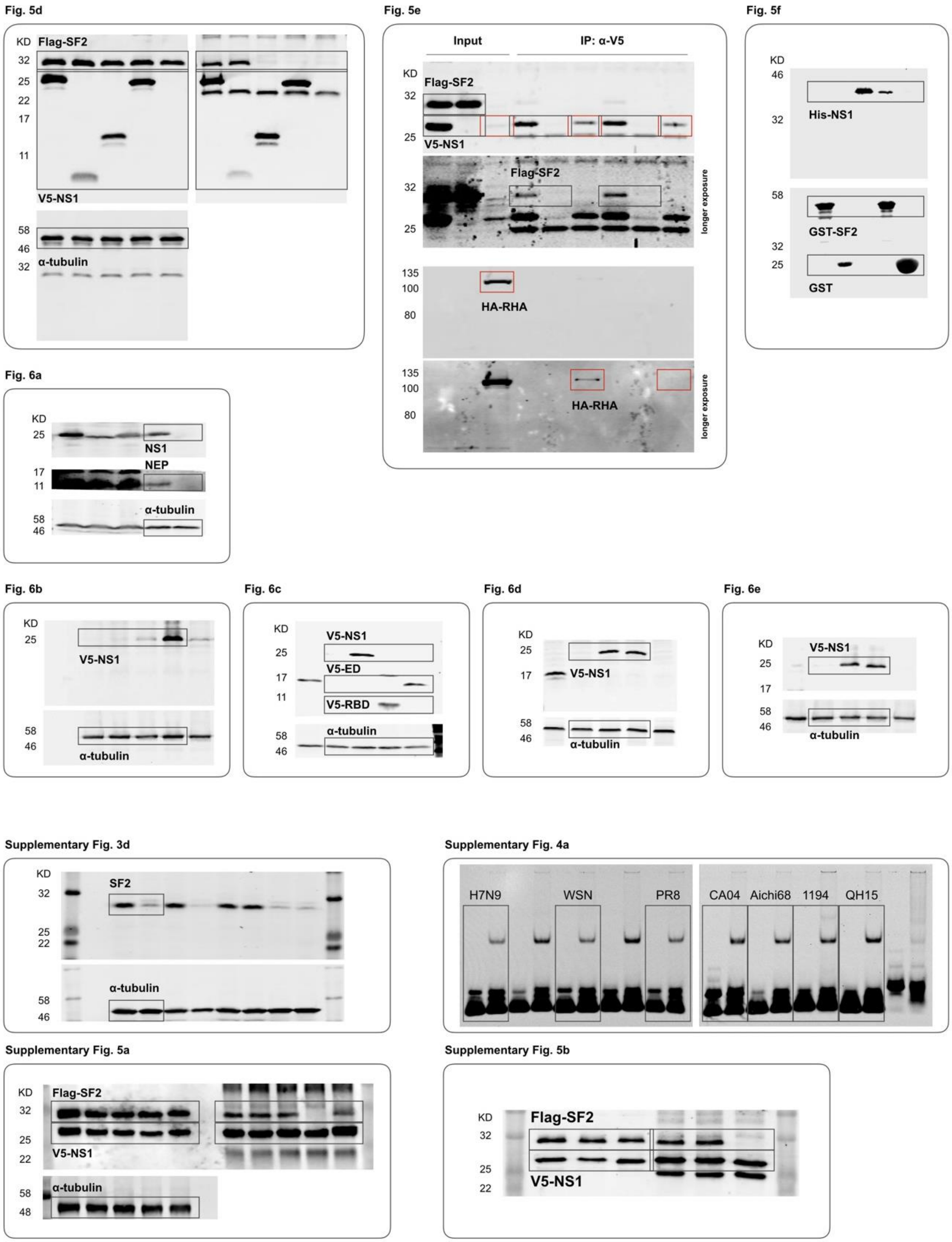

Supplementary Figure 8. Full blots and gels.

Supplementary Table 1 Primers and probes

**Primers for qPCR**	
Name	Sequence
α-tubulin-F	CACTCTGATTGTGCCTTCATGG
α-tubulin-R	CGAGCTTAGTGTAGGTTGGGCGCTCG
Rpl32-F	AGCTCCCAAAAATAGACGCAC
Rpl32-R	TTCATAGCAGTAGGCACAAAGG
U87 scaRNA-F	ATGGGATCATGGAGCAGCTG
U87 scaRNA-R	TCACACCCATGACTGCCACT
IFN-β-F	GACGCCGCATTGACCATCTA
IFN-β-R	CCTTAGGATTTCCACTCTGACT
H7N9-NS1-F	GGAAGAAGCAGCACTCTTGG
H7N9-NS1-R	TTTCTGTTTGGGAATGAGCA
NS-null-NS1-R	TGTCCACTATTGCTTGTCATC
H7N9-NEP-F	CTGTGTCAAGCTTCCAGGAC
H7N9-NEP-R	GATCTCCCATCCTCATCGCT
NS-null-NEP-F	CTGTGTGAAGCTTCCAGGAC
NS-null-NEP-R	ACTTCTGGCTTAACTGTTCTCTCC
pSMN1-F	ATAAGAATGCGGCCGCATAATTCCCCCACCACCTC
pSMN-BGH-R	CCTCGACTGTGCCTTCTA
qPCR-SMN-exon6-7-F	CTGGCTATTATATGGGTTTC
qPCR-SMN-exon6-8-F	CTGGCTATTATATGGAAATGC
qPCR-SMN-BGH-R	TAGAAGGCACAGTCGAGGCT
H7N9-PB1-F	CCTCAAGGACGTGATGGATT
H7N9-PB1-R	GCCTCTTTCAGCATCCTTTG
H7N9-M1-F	CGCACAGAGACTTGAGGATG
H7N9-M1-R	TGGGTCTCCATTCCCATTTA
H7N9-NP-F	CAGTGAAGGGGATAGGGACA
H7N9-NP-R	CCAGGATTTCTGCTCTCTCG
mIFNb-F	CAGCTCCAAGAAAGGACGAA
mIFNb-R	ACCCAGTGCTGGAGAAATTG
mTNFa-F	ACAGAAAGCATGATCCGCG
mTNFa-R	GCCCCCCATCTTTTGGG
mIL-1β-F	GTGGCTGTGGAGAAGCTGTG
mIL-1β-R	GAAGGTCCACGGGAAAGACAC
mIL-6-F	CCAGAAACCGCTATGAAGTTCC
mIL-6-R	TTGTCACCAGCATCAGTCCC
mGAPDH-F	AAGGTCATCCCAGAGCTGAA
mGAPDH-R	CTGCTTCACCACCTTCTTGA

**RNA oligonucleotides for RNA EMSA**	
Name	Sequence
NS-donor site (DS)	CAGGUAGACUG
NS-acceptor site (AS)	CUUCUCUUCCAGGA
H7N9-NS-37-56	AUACUGUGUCAAGCUUCCAG
H7N9-NS-529-548	GACAUACUGACAAGGAUGUC
H7N9-NS-A540G-529-548	GACAUACUGACGAGGAUGUC
WSN-NS-529-548	GACAUACUGAUGAGGAUGUC
PR8-NS-529-548	GACAUACUGCUGAGGAUGUC
CA04-NS-529-548	GACAUACUUAUGAGGAUGUC
Aichi68-NS-529-548	GACAUACUAUUGAGGAUGUC
1194-NS-529-548	GACAUACUGGUGAGGAUGUC
QH15-NS-529-548	GACAUACUAAUGAGGAUGUC
